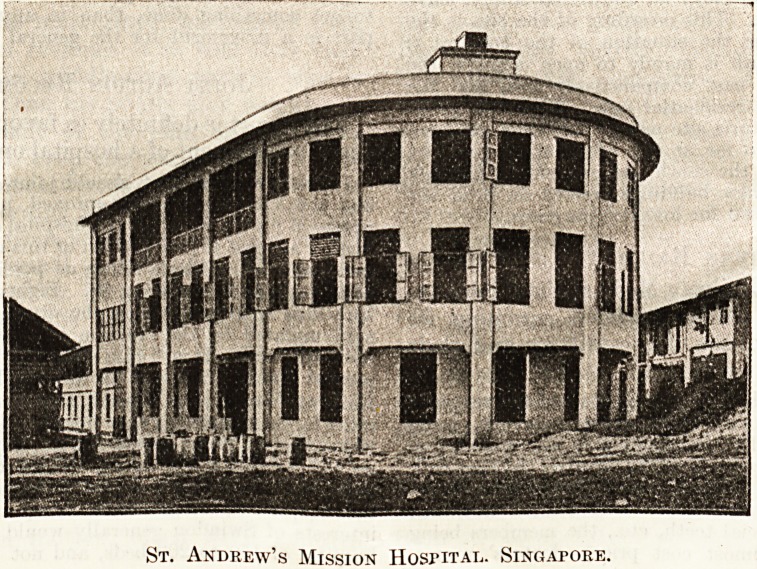# A Dustless and Washable Hospital

**Published:** 1923-10

**Authors:** 


### A DUSTLESS AND WASHABLE HOSPITAL.

### NEW IDEAS IN PAINT AND COLOURS.

AMONG the most interesting examples of recently erected institutions is the St. Andrew's Mission Hospital, Singapore. This building, which has lately been completed, serves the poorer quarters of that town, and is equipped with an operating theatre and an anæsthetic room on the first floor. Completely equipped medical, surgical and maternity wards are also provided, and every effort has been made to follow the best practice of British hospitals. For instance, special departments are installed for the instruction of women in the proper care of children and for training Asiatic girls as nurses; moreover, following the original intention of the Mission from which the Hospital has developed, religious instruction is given.

It will be seen from the accompanying photograph that the building itself is of severe and clear-cut appearance, every consideration of exterior ornamentation being sacrificed to the provision of as much accommodation and equipment as possible. Space is provided for fifty in-patients, and there is also adequate accommodation for out-patients, with quarters for the European and Asiatic staffs, while private wards are provided for paying patients. The architect was Mr. Harry Robinson, of Messrs. Swan & Maclaren, architects, civil engineers and surveyors, of Singapore, and the whole of the work was carried through under his supervision. It was, in fact, due to the energy and enthusiasm displayed by Mr. Robinson and his personal interest in overcoming difficulties that the scheme of erecting the hospital became possible and was ultimately carried through.

Santa Hospital Civil, Bilbao, Spain.

The total cost of the building, including electric lighting, the most modern sanitary and hot and cold water installations, was $111,000. There are no corners in the building to harbour dust, and Messrs. Jenson & Nicholson, Ltd., of Stratford, London, E. 15, manufacturers of the well-known Robbialac Enamel Paint, have provided a special washable enamel for finishing the walls. This is an interesting and important circumstance in view of the discoveries that have been made of the capacity of the brush marks and ridges on ordinary paint to hold disease germs. For this reason many other hospitals are to-day being decorated with enamel paint such as Robbialac, a notable example being that of the Santa Hospital Civil de Bilbao, Spain. The whole of the interior of the immense buildings, shown in the illustration herewith, has been decorated with Robbialac, and it is stated that the surface of this material and its washability entirely obviate the possibility of the accumulation of disease germs.

The selection of wall finishes for hospitals and other institutions, like the subject of colour healing, deserves more attention than it has hitherto received. Considerable advances have, it is true, been made in discovering what are broadly referred to as the soothing and healing influences of colour, but further careful investigation into the subject would be amply repaid, especially for convalescent wards, mental institutions and the like. We cannot afford to neglect any expedient which carries with it possibilities of healing, and nowhere are "sweetness and light" more likely to be appreciated than in hospitals.

St. Andrew's Mission Hospital. Singapore.

## Figures and Tables

**Figure f1:**
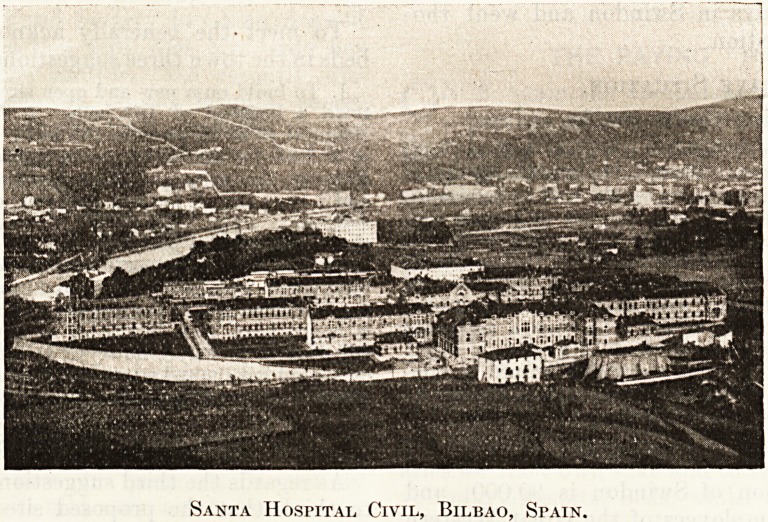


**Figure f2:**